# Mammalian endoreplication emerges to reveal a potential developmental timer

**DOI:** 10.1038/s41418-017-0040-0

**Published:** 2018-01-19

**Authors:** Alberto Gandarillas, Rut Molinuevo, Natalia Sanz-Gómez

**Affiliations:** 1 Cell Cycle, Stem Cell Fate and Cancer Lab, Instituto de Investigación Marqués de Valdecilla (IDIVAL), Santander, Spain; 2 Institut National de la Santé et de la Recherche Médicale (INSERM), Languedoc-Roussillon, Montpellier, France

## Abstract

Among the most intriguing and relevant questions in physiology is how developing tissues correctly coordinate proliferation with differentiation. Endoreplication, in a broad sense, is a consequence of a cell division block in the presence of an active cell cycle, and it typically occurs as cells differentiate terminally to fulfill a specialised function. Until recently, endoreplication was thought to be a rare variation of the cell cycle in mammals, more common in invertebrates and plants. However, in the last years, endoreplication has been uncovered in various tissues in mammalian organisms, including human. A recent report showing that cells in the mammary gland become binucleate at lactation sheds new insight into the importance of mammalian polyploidisation. We here propose that endoreplication is a widespread phenomenon in mammalian developing tissues that results from an automatic, robust and simple self-limiting mechanism coordinating cell multiplication with differentiation. This mechanism might act as a developmental timer. The model has implications for homeostasis control and carcinogenesis.

## Endoreplication

**Fig. 1:**
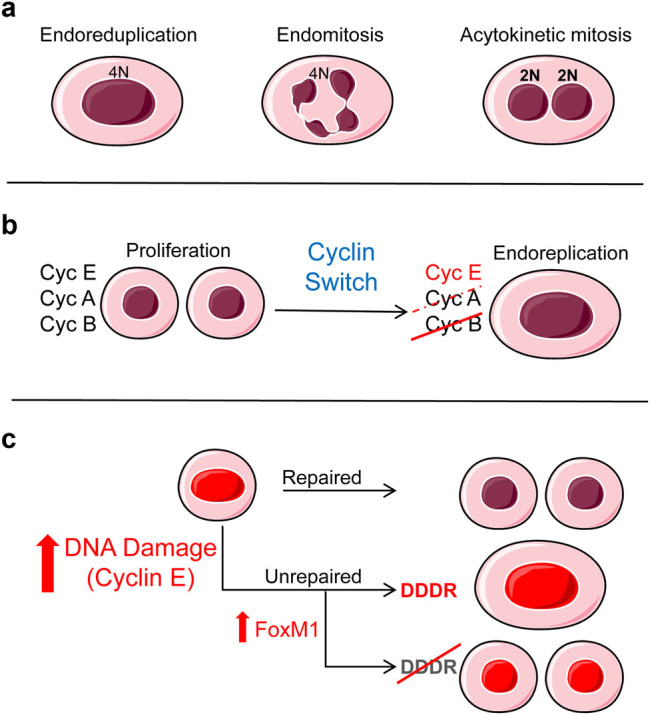
**a** The three main cell products of endoreplication upon: endoreduplication, a single polyploid nucleus; endomitosis, a lobulated polyploid nucleus; and acytokinetic mitosis, two (or more) nuclei. **b** The cyclin switch driving proliferation into endoreplication. During endoreplication, expression of Cyclin B or Cyclin A ceases, while expression of Cyclin E is maintained. **c** The G2 or mitosis checkpoints block cell division and induce endoreplication in response to irreparable DNA damage caused by cell cycle stress, for instance, upon ectopic Cyclin E. The DDDR triggers differentiation, thus suppressing cell divison. In keratinocytes, overexpression of the global mitotic regulator FoxM1 allows damaged cells to continue to divide, thus promoting genomic instability. References within the main text

The definition of endoreplication is somewhat controversial and there is not a general consensus among authors. The associated nomenclature is often confusing, as the mechanisms are very diverse. Endoreplication in a broad sense is defined by some authors as the general phenomenon by which cells undergo DNA replication in the absence of subsequent cell division [1]. According to this definition, three main forms are possible (Fig. 1A): endoreduplication or endocycles (absence of complete mitosis), endomitosis and acytokinetic mitosis (or cytokinesis failure). Endoreduplication is very common in plants [2, 3] and is often known as endocycles in flies [1, 4]. During endoreduplication, the nucleus replicates its DNA without division, becomes large and polyploid and can produce, or not, polytene chromosomes [2, 4, 5]. This can occur via mitosis bypass (without metaphase) or mitotic slippage (with metaphase). During endomitosis the nucleus does not complete division and becomes lobulated. This is typical of mammalian megakaryocytes [6]. In acytokinetic mitosis, the cell achieves karyogenesis by nuclear division, but fails cytokinesis (for some authors this is another form of endomitosis), and the result is a binucleate cell. This is well known in the hepatocytes of the liver [7]. However, some authors make use of the term endoreplication only to refer to endoreduplication or endocycles [4]. To add to the complexity, different variations can coexist within the same tissue [7, 8].

Regardless of the heterogeneity, in all cases the result of endoreplication is a large polyploid cell. Until a needed nomenclature consensus is found, we here will apply the broad sense of the term endoreplication, for any replication of the genome in the absence of subsequent cell division.

Endoreplication initiates due to a deregulation of the proliferative cell cycle leading to dramatic changes that are still not completely understood, but likely originate the diversity of variants. What makes some tissues undergo preferentially one or another form of endoreplication is largely unclear. The most common change leading to endoreduplication or endocycles (lack of karyogenesis) is a switch in the main regulators of the cell cycle, the complexes cyclins/cdks. This switch results in inactivation of the mitotic kinase cdk1 [1, 4–6, 8–11]. In mammals and flies, mitotic cyclins A and B, or just Cyclin B are inactivated, whereas DNA replication Cyclin E persists (Fig. 1b). As a consequence, cells accumulate rounds of DNA replication and growth without division. A similar regulation of the corresponding analog molecules has been found in plants [4, 9]. In addition to the cyclins, regulators of G2, mitosis or cytokinesis, play a role in endoreplication (reviewed in refs. [1, 2, 5, 7, 12]). Molecules controlling the cytoskeleton, the cell division furrow or the cytokinetic actomyosin ring such as RhoA, play a role in endomitosis and binucleation. For instance, differential inhibition of RhoA by downregulation of specific guanine-exchange factors drives endomitosis and subsequent polyploidisation in megakaryocytes [13]. RhoA or cell cycle transcription factors E2F also are involved in cytokinesis failure in hepatocytes (reviewed in ref. [7]). Aurora and polo-like kinases are involved in the spindle assembly checkpoint (SAC) and their deregulation can lead to cytokinesis failure by chromosome missegregation [12]. Aurora B also delays cytokinesis in response to chromosomal defects [14]. These processes and regulations seem to overlap in the control of endoreplication.

The goal of this essay is not to dissect or to review the increasingly complex regulation of endoreplication in the different systems. Regardless of the cell cycle defect, in endoreplicating tissues the homeostatic limiting factor must be within the control of mitosis and cytokinesis. Our aim here is to propose that endoreplication is part of a key automatic control of homeostasis coordinating proliferation with differentiation in self-renewal, expanding, or regenerating tissues.

## Functions of endoreplication

Mammalian endoreplication still often remains as a disregarded odd phenomenon of excentric cells. However, in organisms where this has been carefully studied, it is known to accomplish important homeostatic functions. Endoreplication is typical of cells that perform a specialised function and lose the capacity to divide (i.e., terminal differentiation). These cells commonly are large and produce large amounts of RNA and proteins. This has been extensively studied in plants and flies. For instance, macroscopic plant trychomes are unicellular *hair* OR 'hair' displaying a high number of genomic copies [15]. In these organisms, endoreplication is known to contribute to cell number and organ size control [4, 9, 15]. In *Drosophila*, the formation of large polyploid cells [16] contributes to epidermal wounding and is crucial for the maintenance of the blood-brain barrier during larval brain development [17]. Endoreplication was also proposed as a way to increase the number of gene copies when the cell needs to syntesise large amounts of protein [3, 8]. In *Arabidopsis thaliana*, it controls cell fate maintenance [18].

In mouse, endoreplication has been well studied in trophoblasts, where it is essential to reproduction [11, 19, 20]. In megakaryocytes, disruption of ploidy reduces the production of platelets [21]. Polyploidisation of heart muscle cardiomyocytes seems to facilitate cardiac muscle contraction after myocardial infarction [22]. In human, endoreplication has long been known to occur in megakaryocytes (eventually producing platelets) [6], hepatocytes [7], or endometrium [23]. In last years epidermis [8], heart [24, 25], and mammary gland [26] have been incorporated to this list. Evidence has also been reported in vascular smooth muscle upon hypertension [27], renal podocytes upon glomerulosclerosis [28], uterine smooth muscle during pregnancy [29], and retinal pigment epithelium [30]. Mammalian endoreplication is attracting increasing attention. What seemed to be an exception is slowly becoming a rule. However, the mechanisms coordinating proliferation with endoreplication are unclear.

Rios et al. [26] have recently revealed that the mammalian mammary epithelium becomes binucleate at lactation. Whether some of the nuclei of these binucleate cells undergo endoreduplication and become polyploid is yet to be elucidated. Nevertheless, this constitutes another case of mammalian endoreplication in the broad sense that we use here. The report is particularly interesting to us for two reasons. First, the authors study the consequences of inhibiting the phenomenon on tissue function. They report a drop in milk production when binucleation is inhibited. This might be due to a lesser gene copy number or a less efficient gene expression. Second, this case provides a neat example of a tissue that becomes binucleate at expansion, not at regeneration. This is what we aim to discuss here: the lactating mammary gland becomes binucleate upon an enormous phase of cell multiplication and tissue expansion. The question then is how and when highly proliferative cells block cell division and initiate milk differentiation.

## The DNA damage-induced differentiation response: translating cell growth stimuli into differentiation

Both regeneration and expansion of a developing tissue involves rapid cell growth, proliferation and differentiation. At some point cell multiplication needs to stop to control organ size and function. Some systems have been demonstrated to undergo programmed cell death, namely apoptosis or anoikis, to regulate the number of cells or the size of the biological structure. However, apoptosis aims to suppress unnecessary or malfunctioning cells. The situation is different when tissues expand to accomplish a specialized function, such as protein production, organ barrier, or generation of hard structures.

Human epidermis is a continuously developing self-renewal stratified epithelium. In epidermis, the number of cells generated in the proliferative basal layer must equal the number of cells detaching from the surface of the skin. Even in hyperplastic conditions, such as psoriasis, an excess of differentiation (hyperkeratosis) accompanies increased proliferation. Some mechanisms must ensure that cell cycle hyperactivation does not result in dysplasia or neoplasia. It is thought that soluble factors or intercellular interactions Cross Talk in epidermal basal and suprabasal cells. While clearly this type of regulation exists, we speculate that relying tissue homeostasis only on a complex regulation by soluble or membrane factors increases the risk of malfunctions. Homeostasis must need additional automatic cell-autonomous, self-limiting mechanisms in case things go wrong, some kind of cellular *airbag* OR 'airbag'.

By studying the mechanisms leading to endoreplication in human keratinocytes, we found that cell cycle stress causing DNA damage leads to terminal differentiation and polyploidy [31]. This includes DNA replication stress by oncogenic alterations such as overexpression of MYC or Cyclin E and inhibition of tumor suppressor p53 or genotoxic drugs (Fig. 1c) [8, 31–33]. This DNA damage-differentiation response (from here on, DDDR) likely occurs via induction of G2 or mitosis checkpoints, since merely inhibiting the G2/M transition triggers terminal differentiation. We hypothesized that this might be a simple and very efficient anti-oncogenic mechanism: replication stress caused by loss of cell cycle control would, via terminal differentiation, suppress further proliferation of damaged cells. If this is the case, epidermoid cancer would require alterations in mitosis control in addition to the deregulation of the cell cycle. We have recently obtained evidence for this hypothesis. For instance, well-differentiating carcinoma cells contain alterations in the DDDR, and completely suppressing this response rendered them highly tumorigenic in vivo [34]. In addition, overexpression of the global mitotic regulator FOXM1, frequently amplified in epithelial cancer, allows keratinocytes to further divide in spite of high DNA damage, thus promoting genomic instability (Fig. 1c) [35, 36]. These observations further suggest that the DDDR exerts a protective role that needs to be broken in order for carcinogenesis to progress.

Although a DDDR has been scarcely studied, other authors have reported evidence for this phenomenon. Puri et al. in 2002 [37] proposed the existence of a differentiation checkpoint induced by genotoxic stress inhibiting myogenesis in instable cells. However, other findings report differentiation of cell lineages associated with genotoxicity. Loss of genomic integrity promotes maturation of lymphoid and myeloid lineages [38–40]. Sherman et al. [41] compiled other evidence for this response in neuron and hematopoietic differentiation and suggested that the DDR outcome might depend on the cell type, the degree of damage, or the differentiation state. To note, ionizing radiation (IR) or inhibition of the DNA-repair signaling protein ATM provokes terminal differentiation of melanocyte precursors in mice [42]. IR also promoted mouse astrocytic differentiation [43].

The DDDR might constitute a widespread automatic mechanism to cleanse the tissue of precancerous cells. This is of particular importance in self-renewal systems involving continuous proliferation where apoptosis is not instrumental. Recent reports have shown that DNA damage via differentiation can limit hematopoietic self-renewal [39] and leukemic cancer [40].

How DNA damage drives cells into apoptosis, cell cycle arrest, senescence, or differentiation remains intriguing and is subject of active research. Changes in cell cycle regulators must have a pivotal role here. Tumor suppressors Rb and p53 converge to control the cell cycle. P53 through its transcriptional targets such as p21CIP inhibits DNA replication cdk2 and the G1/S transition, or mitotic cdk1 and the G2/M transition [12]. Loss of p53 often leads to polyploidy. Niculescu et al. [44] suggested that whether p21CIP induces cell cycle arrest in G1, or mitosis arrest and endoreplication, depends on the status of Rb, whose inactivation allows cells to undergo cell cycle progression. Of interest, p21CIP by inducing endoreplication has been shown to protect myeloid or trophoblast cells from apoptosis [45, 46]. Consistently, the inhibition of cdk1 during mitosis arrest by the SAC has been proposed to drive mitotic slippage and endoreplication instead of apoptosis [47]. Regardless of the mechanisms driving one cell fate or another in response to DNA damage, the final outcome appears to support the physiological needs. Endoreplication occurs in tissues that require large cells to fulfill a specialised function that would be lost in the event of apoptosis. For instance, hyperactivation of the cell cycle by MYC inhibits erytroid differentiation, destined to produce small anucleate cells, but it enhances megakaryocyte or keratinocyte differentiation resulting in large polyploid cells [31, 45, 48].

Plants and animals both take advantage of endoreplication so as to enable tissue growth upon DNA damage without the deleterious effect of apoptosis. Under genotoxic stress [49], defective chromatin assembly [4], or telomere shortening [50] some cell types activate the DNA-damage response, block at mitosis, and undergo endoreplication. An endoreplication rise has also been described during regeneration (see also ref. [1]) in human cardiomyocytes after acute myocardial infarction [24] or in mice liver following partial hepatectomy [51] or oxidative stress [52]. Interestingly, the liver can achieve regeneration in the absence of cell proliferation due to polyploidisation [51, 53, 54].

## A proliferation timer

The same role of the DDDR in protecting a tissue from cancer should contribute to maintain homeostasis. Rapid rounds of proliferation via cell cycle hyperactivation increase the index of replication errors and diminish the efficiency of DNA repair, causing replication stress [55, 56]. Accumulation of DNA damage triggers the G2 or the mitotic checkpoints and, via the DDDR, might establish a link between rapid proliferation and terminal differentiation. In self-renewal tissues, daughters of stem cells during their natural PROGRAMME undergo a phase of rapid clonal expansion prior to terminal differentiation [57]. Paradoxically, although these cells are actively proliferating, they have a limited capacity of multiplication, as they are committed to undergo terminal differentiation by unknown mechanisms. The DDDR might fulfill this function as these cells lose control of the cell cycle [32, 33]. Cell cycle stress eventually might limit their capacity to divide. We have obtained a large body of evidence that in human keratinocytes this triggers terminal differentiation. Cyclin E is the major drive of DNA replication, and its deregulation is well known to cause DNA damage via replication stress [58] and to drive endoreplication [10]. It is interesting that differentiating keratinocytes strongly accumulate Cyclin E [8, 32] and that its ectopic expression in proliferative cells results in terminal differentiation and polyploidy [32]. It is tempting to speculate that accumulation of S-phase regulators such as Cyclin E due to cell cycle deregulation results in loss of cell division capacity due to irreparable DNA damage (Fig. 1c). Since a G2/M block leads to terminal differentiation, this would explain why keratinocytes in the rapid proliferative phase are committed to differentiate after only four or five rounds of cell division [57]. Therefore, the DDDR would constitute a cell-autonomous automatic limit to proliferation. We question whether this type of mechanism can apply to other mammalian tissues. We propose it can.

**Fig. 2:**
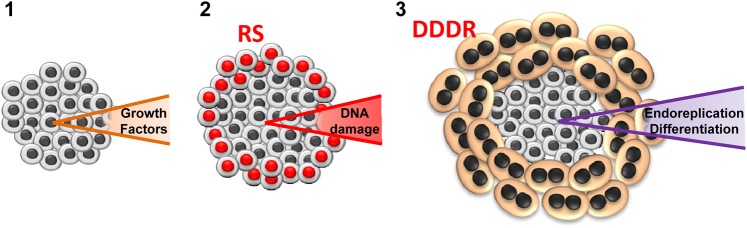
The DNA damage-differentiation response (DDDR) might link proliferation with differentiation in endoreplicating tissues. Cell cycle is hyperactivated by growth factors and cells undergo proliferation (**1**). Active proliferating cells accumulate DNA damage due to replication stress (RS; red nuclei; **2**). The DDDR pathway is activated upon a prolonged G2/M arrest and irreparable cells undergo differentiation and endoreplication (3)

As discussed above, a polyploidy rise has been observed in a diversity of biological systems upon hyperplasia, tissue regeneration, or expansion, conditions in which the level of replication stress is high. These situations require sustained and rapid proliferation that must be tightly coordinated with differentiation. We propose that the DDDR acts as a self-limiting mechanism to time proliferation and link cell multiplication with terminal differentiation in developing tissues, thereby controlling cell number and organ size and function (Fig. 2). The limit to cell proliferation would be imposed by itself due to the replication stress caused during hyperactivation of the cell cycle. Cells not having robust G2 or mitosis checkpoints, not undergoing apoptosis, would terminally differentiate, endoreplicate, and start massive protein production. Terminal differentiation might irreversibly suppress cell division by physical constraint [8]. While this manuscript was under review, Cao et al. have reported that mechanical tension induces binucleation in the growth front of expanding heart explants in vitro [59].

As a result of the DDDR, tissues upon cell cycle hyperactivation would undergo benign hyperplasia (as in skin wound-healing or in the lactating mammary gland). Alterations in the DDDR in addition to cell cycle hyperactivation would be required for tumorigenesis. Therefore, in endoreplicating tissues, the limiting factor in homeostasis and cancer might lie within the G2 and mitosis checkpoints. If the main goal of cells is to most correctly transmit their genetic material to the progeny, then in developing tissues the pathways controlling DNA damage and repair should be tightly linked with the control of post-mitotic differentiation.
